# A Voltage-Sensitive Dye-Based Assay for the Identification of Differentiated Neurons Derived from Embryonic Neural Stem Cell Cultures

**DOI:** 10.1371/journal.pone.0013833

**Published:** 2010-11-04

**Authors:** Richardson N. Leão, Amilcar Reis, Amanda Emirandetti, Michalina Lewicka, Ola Hermanson, André Fisahn

**Affiliations:** 1 Neuronal Oscillations Laboratory, Karolinska Institutet, Stockholm, Sweden; 2 Department of Neuroscience, Karolinska Institutet, Stockholm, Sweden; 3 Developmental Genetics Group, Department of Neuroscience, Uppsala University, Uppsala, Sweden; University of California Merced, United States of America

## Abstract

**Background:**

Pluripotent and multipotent stem cells hold great therapeutical promise for the replacement of degenerated tissue in neurological diseases. To fulfill that promise we have to understand the mechanisms underlying the differentiation of multipotent cells into specific types of neurons. Embryonic stem cell (ESC) and embryonic neural stem cell (NSC) cultures provide a valuable tool to study the processes of neural differentiation, which can be assessed using immunohistochemistry, gene expression, Ca^2+^-imaging or electrophysiology. However, indirect methods such as protein and gene analysis cannot provide direct evidence of neuronal functionality. In contrast, direct methods such as electrophysiological techniques are well suited to produce direct evidence of neural functionality but are limited to the study of a few cells on a culture plate.

**Methodology/Principal Findings:**

In this study we describe a novel method for the detection of action potential-capable neurons differentiated from embryonic NSC cultures using fast voltage-sensitive dyes (VSD). We found that the use of extracellularly applied VSD resulted in a more detailed labeling of cellular processes compared to calcium indicators. In addition, VSD changes in fluorescence translated precisely to action potential kinetics as assessed by the injection of simulated slow and fast sodium currents using the dynamic clamp technique. We further demonstrate the use of a finite element model of the NSC culture cover slip for optimizing electrical stimulation parameters.

**Conclusions/Significance:**

Our method allows for a repeatable fast and accurate stimulation of neurons derived from stem cell cultures to assess their differentiation state, which is capable of monitoring large amounts of cells without harming the overall culture.

## Introduction

A major goal of stem cell therapy is to be able to replace lesioned or degenerated cells and tissue in patients suffering from various neurological disorders. Whereas stem cells and progenitors have been in clinical use for decades in fields such as hematology, there are still major obstacles to overcome before cell replacement in the CNS could become a common clinical practice. With this aim, it is important to increase our understanding of the mechanisms underlying the development of pluripotent (embryonic stem (ES) cells) and multipotent undifferentiated cells into specific types of neurons. Embryonic neural stem cell (NSC) cultures provide a valuable tool to study the fundamental processes of neural differentiation. Differentiation mechanisms in NSC cultures are routinely assessed using immunohistochemistry or gene expression analysis for cell-specific cytoskeleton proteins, voltage-gated channels etc [1], whereas Ca^2+^ imaging and/or electrophysiology are techniques used less frequently [2], [3].

Protein level and gene expression analysis are optimal for quantitative studies but these methods cannot provide direct evidence of neuronal functionality. Calcium imaging and electrophysiology on the other hand can provide direct evidence of neuronal functionality. Electrophysiological techniques in particular are the most informative methods to investigate synaptic, membrane and channel properties in stem cell-derived neurons. However, electrophysiological recording techniques can only be used to study a few cells on a culture plate. This is an even greater problem when studying neurons or neuron-like cells derived from floating ‘eurospheres’[2]. Neurospheres are aggregates of neural progenitors containing a population of NSCs, and often used in NSC research due to their ability of self-renewal and their relative stability [2]. Importantly, cells derived from these neurospheres are often at different developmental stages and hence the process of searching for functional neurons using electrophysiological techniques (e.g. patch clamp or sharp microelectrodes) can be time consuming and result in a deterioration of the health of the cells on the culture plate. Ca^2+^ imaging after bulk loading on the other hand can be used to analyze a greater number of cells simultaneously but the loading with Ca^2+^-sensitive dyes is time consuming and often kills a large proportion of cells on the plate [3]. Moreover, some compounds used in the dye-loading process can alter membrane properties (e.g. DMSO). In addition, Ca^2+^ imaging can only provide indirect evidence of electrical activity. For example, it is not possible to differentiate between mature and immature Na^+^ currents due to the slower changes of [Ca^2+^] in relation to an action potential and also the ‘nertia’of the Ca^2+^ indicators [4]. Voltage sensitive dyes (VSD) have been extensively used in brain slices and *in vivo* [5]. Here, we demonstrate the advantages of using voltage-sensitive dyes (VSD) [6], [7] to detect fully differentiated neurons derived from neural stem cell cultures.

## Materials and Methods

### Embryonic Neural Stem Cell Cultures

Isolation and culture of multipotent neural progenitors, referred to as NSCs, were performed as previously described [8], [9], [10]. Briefly, cortices from rats at embryonic day 15.5 were dissected and mechanically dissociated in a modified serum-free, HEPES-free N2-supplemented DMEM/F12 medium (Invitrogen, Carlsbad, CA, USA). The primary cells were seeded at a density of 1.0×10^6^ cells on 100 mm dishes pre-coated with poly-L-ornithine and fibronectin (Sigma, St. Louis, MO, USA).

The cells were cultured as monolayers throughout the experimental procedures, expanded in human recombinant basic FGF2 (R&D Systems, Minneapolis, MN, USA) at 10 ng/ml every 24 h, and the N2 medium was replaced every 48 h. Generally, experiments were performed after the first passage with cells seeded at a density of 0.2×10^6^ on 60 mm plates and when >90% of the cells displayed nestin expression and typical neural stem cell morphology and <1% of the cells expressed neuronal and glial differentiation markers.

To achieve differentiation into neuronal-like cells, NSCs were treated with the HDAC inhibitor valproic acid (VPA, Sigma, St. Louis, MO, USA) at 1 mM for 7, 14 or 21 days [11]. Alternatively, 10 ng/ml BMP4 and 10 ng/ml Wnt3a (R&D Systems, Minneapolis, MN, USA) were administered every 24 h for 14 or 21 days [10].

Ethical permission for all work including animals and tissue was granted by Northern Stockholm's animal research ethics committee (Norra Djurförsökningsnämnd, permit numbers N109/07 and N79/08).

### Primary hippocampal cultures

Primary cultures of hippocampal neurons were prepared as described previously [12]. All chemicals used in our primary cultures were purchased from Sigma-Aldrich unless stated otherwise. Hippocampi were dissected from 14 days-old C57BL6 mice in ice-cold dissection solution containing glucose/HEPES (10 mM HEPES, 12 mM NaHCO_3_, 137 mM NaCl, 2.7 mM KCl, 5 mM glucose, 1 mM CaCl_2_, pH 7.4). Isolated hippocampi were gently dissociated in a digestion solution containing 0.25% trypsin and 0.02% EDTA (10 min, 37°C) then washed twice with a solution containing trypsin inhibitor (20 mg BSA, 47.6 mg HEPES, 10 mg trypsin inhibitor in 20 ml Dulbecco's Modified Eagle Medium (DMEM; Invitrogen)). After trituration, the suspension was centrifuged at 200 RPM for 2 min and 300 RPM for 3 minutes to separate the tissue debris from dissociated cells. The resulting cell suspension was seeded in 35 mm poly-D-lysine coated petri dishes and cultured with DMEM supplemented with B27 (Gibco) supplemented with 1 mM sodium pyruvate, 26 mM NaHCO_3_, 2 mM L-glutamine, 33 mM D-glucose, 0.2% B27, 10% horse serum. Streptomycin and penicillin were also added (100 U/ml penicillin, 100 µg/ml streptomycin; Gibco). Cultures were kept at 37°C in a humid 5% CO2-containing atmosphere until use in experiments.

### Fluo-4 and VSD Loading, Imaging

Embryonic neural stem cells were maintained in culture medium and then loaded with the calcium-sensitive dye Fluo-4 AM (Invitrogen) or the voltage-sensitive dyes di-1-ANEPPQ (JPW3027) (synthesized and kindly provided by Leslie Loew, University of Connecticut) or RH795 (Invitrogen). For the Fluo-4 loading, cultures were maintained is a Fluo-3 solution (5 µM) in artificial cerebrospinal fluid plus HEPES (ACSF_H_) buffer (in mM: 119 NaCl, 26 NaHCO_3_, 10 glucose, 2.5 KCl, 1 NaH_2_PO_4_, 1.5 MgSO_4_, and 1.5 CaCl_2_) containing pluronic acid (0,02%) and incubated at 37°Cin the dark. The cells were then washed 3 times and incubated for 60 minutes in ACSF buffer in the dark. The cells were maintained in ACSF buffer throughout the experiment. Fluorescence measurements were performed using an Andor DU-860 electron multiplying (EM) CCD camera (Andor, Belfast, Ireland). To minimize noise, the EM gain was adjusted to produce maximum intensities >50% from saturation and the CCD sensor was cooled to −80°C. Images were acquired at 1000 frames per second. The specimen was excited with a 470 nm LED array (Roithner Laser, Vienna, Austria) custom mounted on an upright microscope and the emission light was filtered with a 505 nm low-pass filter. For the experiments in primary hippocampal cultures we used a 200 W metal-halide lamp (Prior Scientific) with the appropriate emission filters (Chroma). Images were captured using custom-made software (‘iaFluor’ running in Labview (National Instruments, USA). Emission intensity was adjusted in order to generate signals above 2 times standard deviation of the noise.

### Live/dead assay

After one passage (p1), NSCs were seeded at a density of 5×10^4^ in 35 mm plates pre-coated with poly-L-ornithine and fibronectin (Sigma, St. Louis, MO, USA) and expanded in N2 media until >60% confluence was achieved. The NSCs were then differentiated with 1 mM VPA for 10 days. The voltage sensitive dyes JPW3027, RH795 and the calcium-sensitive dye Fura-2 (Invitrogen) dye were applied separately to each plate containing differentiated cells. Fluo-4 could not be used in this assay as both emission and excitation spectra coincided with the live/dead assay spectra. Cell viability was assessed using a Live/Dead kit (Cat: #04511, Sigma) according to the manufacturers standard protocol.

### Electrophysiology and Immunohistochemistry

For current-clamp recordings, glass pipettes filled with a K^+^-gluconate internal solution (in mM: 17.5 KCl, 122.5 K-gluconate, 9 NaCl, 1 MgCl_2_, 3 Mg-ATP, 0.3 GTP-Tris, 1 HEPES and 0.2 EGTA). Pipettes had resistances ranging from 4 to 6 MΩ. Only recordings with <15 MΩ series resistance were used for analysis. Data was acquired using a patch-clamp amplifier (Axopatch 200B, Molecular Devices, USA), low-pass filtered at 10 kHz, digitized at 20K samples/second using WinWCP (Dr. John Dempster, Strathclyde University, UK) and analyzed using Matlab (Mathworks, USA) and R (http://cran.r-project.org/). Data is presented as means ±SEM and the *t*-test was used to compare differences between means.

In some experiments, cultures were used for immunohistochemistry after patch-clamp recordings. After the electrophysiology experiment cultures were fixed with 4% paraformaldehyde for 20 min. followed by quenching in 10% donkey serum in PBS (in mM: 137 NaCl, 4.3 Na_2_HPO_4_, 1.47, KH_2_PO_4_; pH 7.4). Cultures were then incubated for 20 min. in 1/500 anti-Map2A/B antibodies (Cat.: MAB3418, Millipore) in PBS. Followed by 3×10 min washes in PBS and secondary antibody staining (CY2 anti mouse, Jackson).

### Dynamic-Clamp

In order to assess the functional effects of different action potential (AP) kinetics in VSD imaging in cultured cells, we have simulated an ‘mmature’and a ‘mature’ Na^+^-current (*I*_Na_) in cultured cells using the dynamic clamp technique. This method was implemented on a second computer running a Linux kernel modified by the Real Time Application Interface for Linux from the Politecnico di Milano Institute - Dipartimento di Ingegneria Aerospaziale (RTAI, http://www.rtai.org) and custom-made software that reads membrane voltage and generates current commands at a 40 kHz refresh rate using a DAQ card (National Instruments) and drivers from the Linux Control And Measurement Device Interface (COMEDI, http://www.comedi.org). Our two *I_Na_* model followed a Hodgkin and Huxley notation: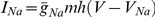, where 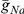 is the maximal Na^+^ conductance, *V* is the membrane voltage and *V_Na_* is the Na^+^ reversal potential. The evolution variables *m* and *h* were numerically solved using the following equation:
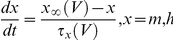



### 2D Finite Element and Passive Cell Model

We used the finite element technique to model the electric field across two electrodes in a culture plate (Johnson, 1995). In this technique, a field region is divided into small discrete elements or nodes (mesh) to facilitate the calculation of differential equations in systems with complex geometry. The electric potential distribution across a coverslip using a bipolar electrode can be obtained by solving the Laplace equation: 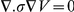 (where 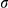 is the conductivity and 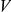 is the electric potential). We used an arbitrary distribution of passive cells of various shapes to simulate the effect of extracellular stimulation. Meshes were generated and refined automatically in Matlab and the PDE toolbox (Mathworks) using the Delaunay triangulation algorithm [13]. The poles of extracellular electrodes were either placed in the vicinity of the cells or with a negative pole nearer and the positive pole farther away from the cells. The effect of the stimulation pulse on membrane voltage was calculated using equations described previously [14].

## Results

### Extracellular JPW3027 produces a more specific labeling of cell body and processes

**Figure 1:**
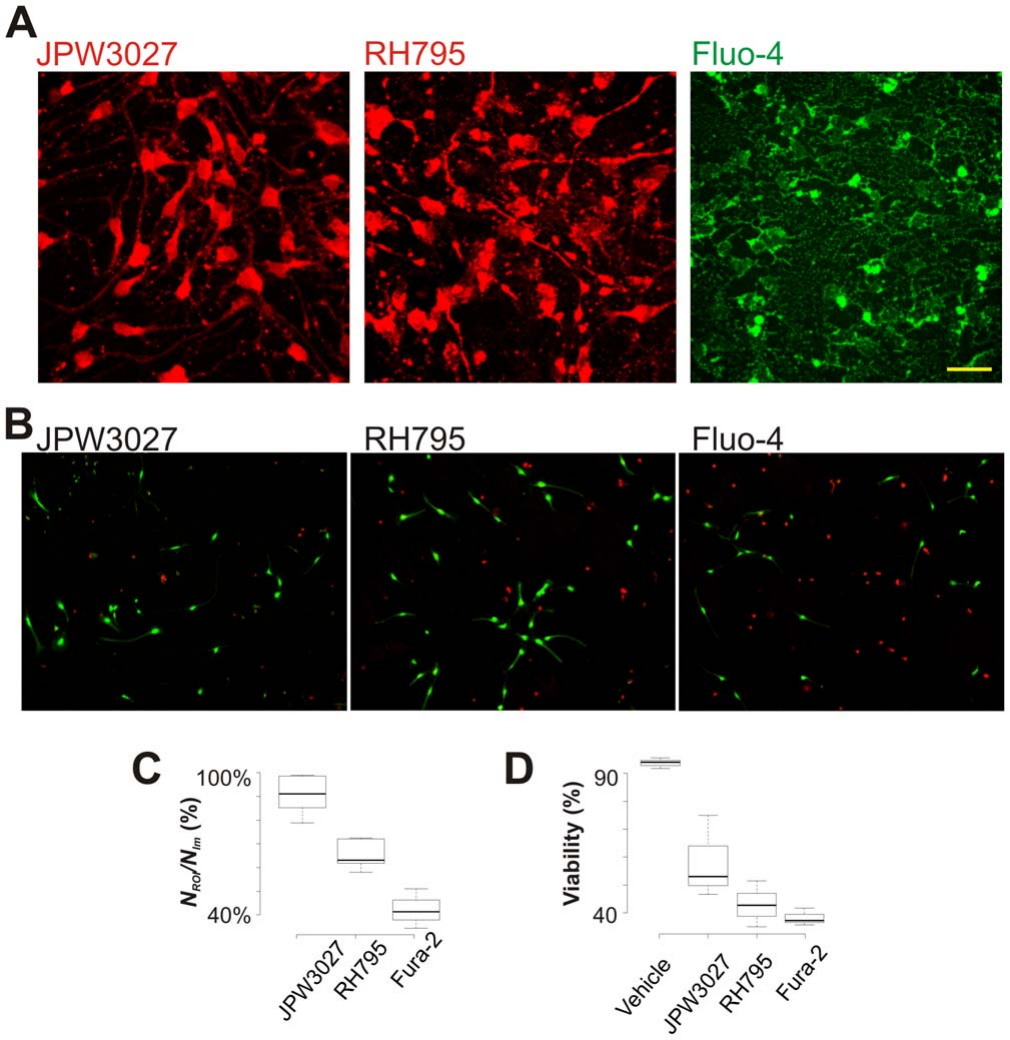
Extracellularly-applied JPW3027 produces specific labeling of cell body and processes. A. Examples of confocal sections of a coverslip stained with JPW3027, RH795 and Fluo-3. Scale bar  = 50 µm. B. Examples of fluorescence photomicrographs of ‘live/dead’ assays used to estimate cell death caused by different dyes (JPW3027, RH795 and Fura-2). C. Summary histogram showing the percentage of bright pixels that are contained within a cell indicating quality of labeling of cell body and cell processes following the loading with different dyes. D. Summary histogram showing the percentage of viable cells following the loading with vehicle or different dyes.

We first tested the ability of VSDs to label cellular membranes without marking debris or the coverslip coating, which would result in increased background fluorescence and, therefore, decreased quality of the voltage sensitive signal. For these experiments we used embryonic multipotent NSC cultures derived from mid-gestation rat embryonic cortex and expanded as monolayers in FGF2 [8], [9], [10], [15]. The NSCs were differentiated with a protocol based on treatment with the histone deacetylase inhibitor valproic acid (VPA), which generates increased number of cells with neuronal-like morphology and marker expression a vast majority of which, however, are electrically inactive (see further below). Thirteen coverslips containing embryonic NSC cultures differentiated with VPA (7, 14 and 21 days) were labeled with JPW3027 (*n* = 6), RH795 (*n* = 5) and the Ca^2+^ indicator Fluo-4 (*n* = 3). Coverslips were examined in a confocal microscope with the same pinhole and laser power settings and detector gain and offset were adjusted in order to produce 8-bit images in which 0.5% of the pixels had intensities *F* = 0 and another 0.5% with *F* = 255 (saturated). To quantify specific vs. non-specific cell labeling, we first outlined cell bodies and processes manually and counted the number of pixels (*N*_ROI_) within the outlines that showed intensities greater than the mean total image intensity (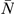) plus two times the standard deviation of the total image intensity (*F>* 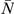
+ 2σ*_N_*). We then counted the number of pixels in the whole image (*N*_im_) with *F>* 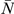
+ 2σ*_N_*. Hence, the ratio of *N*_ROI_/*N*_im_ indicates the percentage of bright pixels that are contained within a cell. The *N*_ROI_/*N*_im_ ratio was equal to 90.6±3.3% while the *N*_ROI_/*N*_im_ ratio after RH795 was equal to 65.5±2.9% and 42.3±3.8% (*n = *14, *p<*0.01, ANOVA, Fig. 1A, C). To estimate cell viability after dye loading, we performed a live/dead assay in cells after 10 days of VPA differentiation. Twelve coverslips with cells were loaded the day before the analysis with either ACSF_H_ (vehicle), JPW3027, RH795 or Fura-2 (we used Fura-2 instead of Fluo-4 as the latter's excitation/emission wavelengths coincides with the wavelengths of our live/dead assay). Cell viability was equal to 93.7±1.1% for coverslips loaded with the vehicle, 59.2±8.5% for JPW3027, 43.1±4.7% for RH795 and 38.3±1.8% for Fura-2 (*n* = 12, *p<*0.01, ANOVA, Fig. 1B, D). When comparing differences in cell viability between Fura-2 and JPW3027 and between RH795 and JPW3027 p values were *p* = 0.04 and *p* = 0.10, respectively (Fig. 1D).

Passive membrane properties of cells loaded with JPW3027 were not significantly different from control cells. In coverslips containing cells differentiated for 2 weeks using VPA, cell capacitance was equal to 18.3±4.2 pF in control and 17.5±5.6 pF in the presence of JPW3027 (not significant, *n* = 16). Membrane potential in 2-week differentiated cells (VPA) was equal to −29.7±5.3 mV in control and −28.9±4.4 mV in cells loaded with JPW3027 (not significant, *n* = 16). Input resistance in control cells was equal to 3.3±0.6 GΩ and 3.2±0.8 GΩ in the presence of JPW3027.

**Figure 2:**
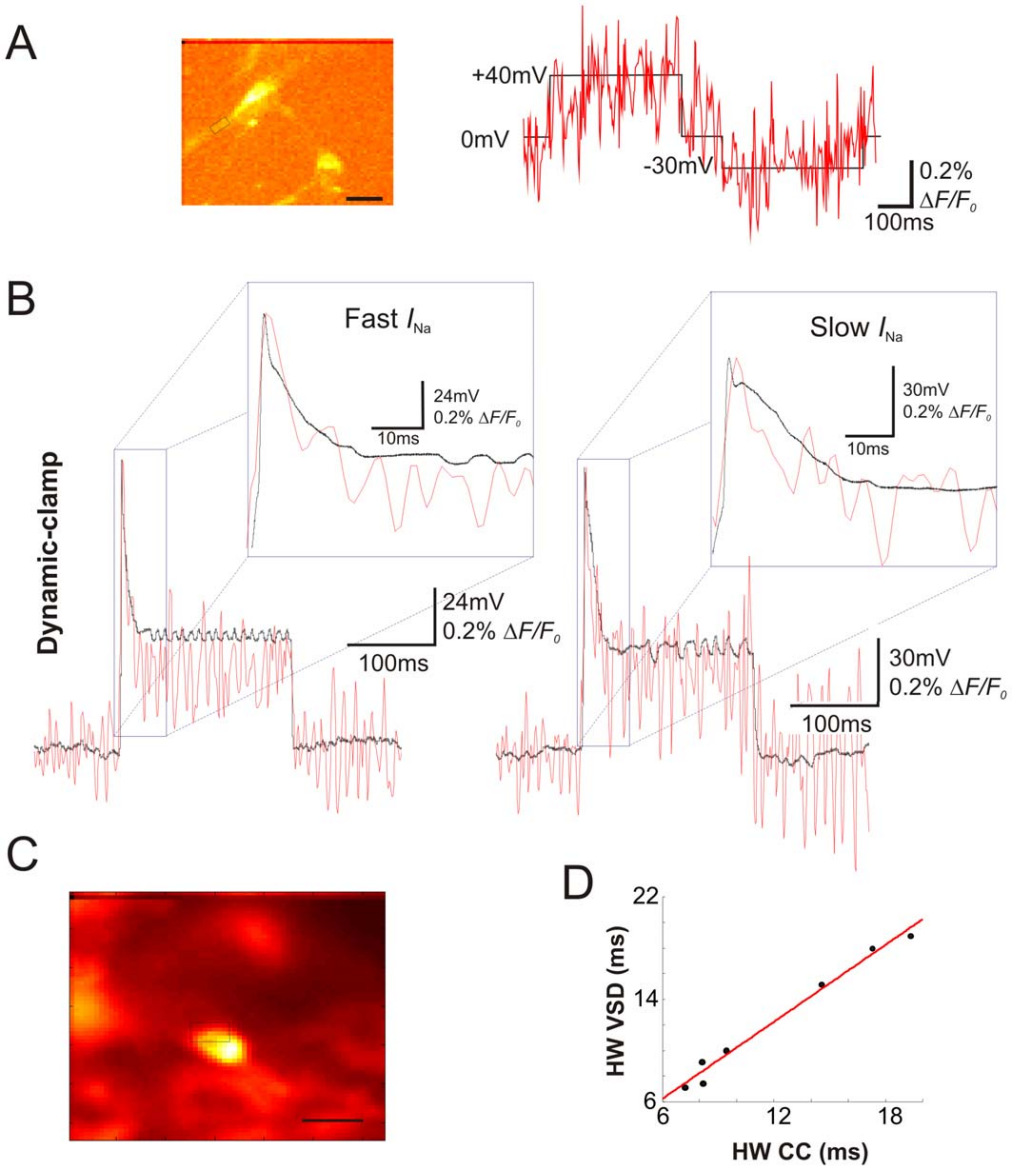
VSD can distinguish fast versus slow AP kinetics. A. Single sweep fluorescence change (Δ*F*/*F*_0_) of a cell loaded with JPW3027 (extracellular) in response to voltage steps of 40mV and −30mV. Scale bar  = 50 µm. B. Example of current-clamp recordings of a cell when a ‘fast’ and ‘slow’ simulated *I*_Na_ (dynamic-clamp) was added to the cell and the concomitant fluorescence response (single sweep, averaged from 40 pixels). C. Single-frame of the image series used in A showing the region in which the fluorescent signal was measured (small rectangle). Scale bar  = 50 µm. D. Relationship between the ‘AP’ half-width measurements from current-clamp and fluorescent traces.

The voltage sensitivity of extracellularly applied JPW3027 was assessed using fast imaging and voltage-clamp recordings. Fluorescence changes were measured as the ratio of fluorescence change (Δ*F*) over the initial fluorescence intensity (*F*_0_). A +40 mV voltage-step produced Δ*F*/*F*_0_ ranging from 0.12% to 0.41% (measured from a perisomatic region containing around 20 pixels and averaged from 4 repetitions; average  = 0.2±0.05%, *n* = 6, Fig. 2A). If maximal LED (emission) intensity was used, a +40 mV step produced a mean Δ*F*/*F*_0_ response equal to 1.93±0.4% (*n* = 6). Even greater fluorescent changes were observed with a metal halide lamp (see experiments in primary hippocampal cultures below). However, under those conditions the noise of the fluorescence signal was considerably higher.

### VSD fluorescent changes translate precisely the kinetics of action potentials

AP kinetics are an important indicator of the developmental stage of a neuron [2]. However, due to the speed of the events underlying the generation of APs, Ca^2+^ imaging assays fail to mirror fast AP dynamics [16]. Hence, we assessed the possibility of using VSD to analyze AP dynamics. Using dynamic clamp, we simulated a fast and a slow current in cells that did display any native *I_Na_* (differentiated for 14 or 21 days with VPA, loaded with JPW3027; Fig. 2B, C, D). A 100 pA current step was used to trigger an artificial AP. The half-width (HW) of the fluorescence signal (after fitting a single exponential to the re-polarization trace) and the current clamp recording produced by the artificial AP showed a strong relationship *r* ^2^ = 0.98 (*p = *0, *n* = 7, Fig. 2D).

### VSD fluorescence can be used to detect active neurons

**Figure 3:**
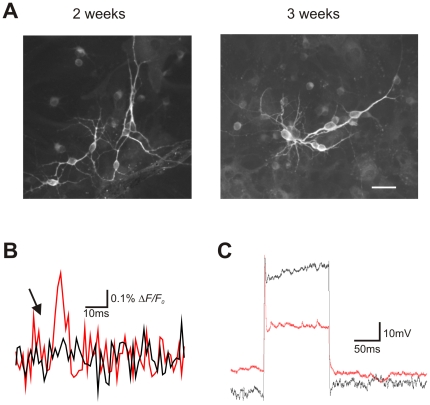
Single APs can be detected using extracellularly-applied JPW3027. A. Examples of MAP2A, B staining of embryonic neural stem cell cultures differentiated during two and three weeks. Scale bar for both images  = 50 µm. B. Fluorescence response of two cells on a cover slip after electrical stimulation (red and black traces show cell with and without a detectable response, respectively). C. Current-clamp recordings of the cells used in ‘B’ (same color coding for the traces).

Morphological clues are not infallible in determining which neurons in a plate are active (i.e. capable of generating APs). In coverslips differentiated over two and three weeks using combined treatment with BMP4 and Wnt3a [17], almost no visible difference in morphology was observed among the generated neuronal-like cells nor in the detection of the late dendro-somatic neuronal marker MAP2A,B (Fig. 3A). In coverslips containing NSCs differentiated by co-treatment of BMP4 and Wnt3a for two weeks we found no active neurons after recording from 15 cells using the patch clamp technique. Only 3 out of 20 cells showed some form of immature APs after current injection in cultures differentiated for three weeks (example in Fig. 3C). APs (like the ones in Fig. 3C) could be blocked by the application of 1 µM TTX (*n* = 4).

Membrane properties of cultured cells expressing MAP2A/B do not resemble properties of functional neurons. Mean resting potential and input resistance for NSC differentiated for one week (with VPA) was equal to −10±3 mV and 1.6±0.3 GΩ (*n* = 5), −19±5 mV and 0.93±0.4 GΩ (*n* = 4) for two-week differentiated cells and −32±4 mV and 0.8±0.2 GΩ (*n* = 5) for 3-week differentiated cultures. Treatment of BMP4 and Wnt3a produced cells with mean resting potential and input resistance equal to −22±3 mV and 1.1±0.2 GΩ (two-week differentiation, *n* = 5) and −35±5 mV and 0.8±0.3 GΩ (two-week differentiation, *n* = 5).

Using a bipolar tungsten electrode with a ∼1.5 mm distance between the uncoated tips (uncoated surface area ∼2500 µm^2^) we applied 1–10V stimuli while acquiring fluorescence in cultures labeled with JPW3027. Cells that produced Δ*F*/*F*_0_ peaks also showed APs when patched (12 out of 12 cells, 3-week BMP4 and Wnt3a-treatment culture; Fig. 3B, C) while the majority of cells in which no fluorescence peak was observed showed no AP during current-clamp recordings (9 out of 12 cells; Fig. 3B, C). Interestingly, even in cultures differentiated for three weeks, all AP-capable cells displayed immature APs (Fig. 3C) that could be suppressed with 1 µM TTX (*n* = 4, data not shown). These results suggest that JPW3027 can reliably detect electrically active neurons derived from stem cell cultures and distinguish such cells from morphologically indistinguishable non-active cells.

**Figure 4:**
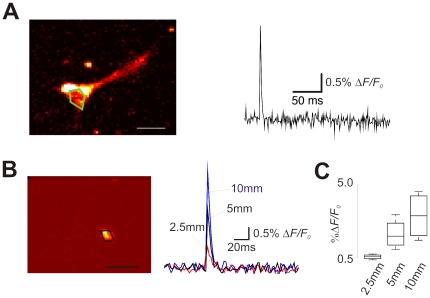
Moving the cathode away from the cell causes greater fluorescence changes. A. Fluorescence response (measured at the area outlined in green) of a cultured hippocampal neuron (left panel, scale bar  = 20 µm) to an electric stimulus. B. Right, Fluorescence response of a glia cell (shown on the left) after electrical stimulation. The anode position was maintained fixed at −2.5 mm (in reference of the cell position at 0 mm) while the cathode was placed at 2.5 (red trace), 5 (black trace) and 10 mm (blue trace). Stimulus amplitude was also fixed (1V, 5 ms). The traces are the averages of 20 repetitions. Left, single-frame of the image series of a glia cell (astrocyte) showing the region in which the fluorescent signal was measured (outlined in black). Scale bar  = 20 µm. C. Summary of fluorescence change at different cathode positions.

We further evaluated the ability of detecting firing cells in cultures by labeling primary hippocampal cultures with JPW3027. These cultures contained both neuronal and non-neuronal (glial) cells. A 200 W metal-halide lamp was used to excite the dyes in these experiments. A 1 V, 5 ms stimulus pulse produced a mean Δ*F*/*F*_0_ of 3.6±0.4% (*n* = 4, Fig. 4A and Movie S1); however, noise and bleaching was considerably higher when compared to LED excitation (data not shown). The bipolar stimulation electrodes were placed equidistantly from the neuron (the neuron was 2.5 mm away from the anode and 2.5 mm away from the cathode). We also analyze the effect of stimulation electrode positioning on the emission of the JPW3027. These experiments were conducted on non-neuronal (glial) cells in order to avoid interference of the non-linear electrical stimulation-to-fluorescent emission relationship exhibited by neurons with the experimental aim. The anode was placed at a −2.5 mm position in relation of a glial cell (astrocyte; set at position 0) while the cathode was placed at the opposite direction in different positions (2.5, 5 and 10 mm). Moving the cathode to the positions 2.5, 5 and 10 mm produced mean Δ*F*/*F*_0_ (metal halide illumination) of 0.5±0.7%, 1.9±0.4% and 3.1±0.7%, respectively (*n* = 4, *p* = 0.02, ANOVA, Fig. 4B, C).

### A finite element model of the coverslip and electrodes can be used to optimize stimulation parameters

**Figure 5:**
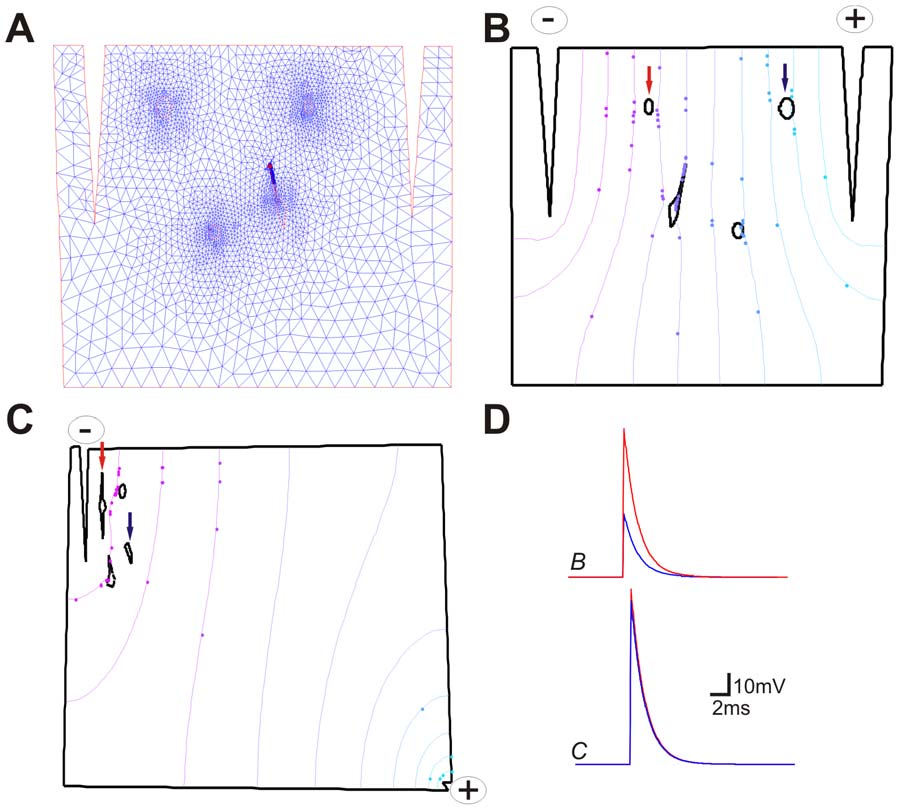
Simple finite element models can be used to determine the electrical potential in distributed cells. A. An arbitrary geometry of a coverslip containing four cells flanked by two stimulating electrodes (+ and −) with a mesh produced by the Delaunay triangulation algorithm. B. Isopotential lines across the two poles. C. Isopotential lines in another model in which the (−) electrode was placed distant from the (+) electrode. D. Membrane response of two identical passive cells placed in different locations of the coverslip in B and C. Red and blue traces correspond to the cells marked with the red and blue arrows in B (top traces) and C (bottom traces).

A problem of using extracellularly applied electrical stimulation to trigger APs is the difficulty to assure that the cells surrounded by the electrodes are reaching AP-threshold after stimulation. This problem is minimized in a monolayer culture as the geometry can be precisely modeled using simple phase-contrast micrographs. We used arbitrary (and simplified) geometries of a monolayer of cells to demonstrate that a 2D finite element model can serve as a useful tool to determine the voltage across the neuronal membrane after electrical stimulation. Arbitrary geometries for cells and plate were used to demonstrate the principle (Fig. 5A). For 2D finite element model calculations, we used the standard partial differential equations (PDE) toolbox of Matlab. PDE were obtained and solved for each node (triangle) of the mesh (Fig. 5A). Nodes were computed using the Delaunay triangulation algorithm. Similar to the experimental results described above and shown in Fig. 4B, our finite element model shows that when electrodes are placed close together (Fig. 5A, B), the voltage distribution across the electrodes can cause variable voltage deflections in different cells (Fig. 5A, B). However, if the (+) electrode is far from the patch of cells, cells are enclosed in an almost isopotential domain and the electrical stimulation is likely to affect the cells homogeneously (Fig. 5C).

## Discussion

In this study we describe the application of the fast voltage probe JPW3027 to the detection of action potential-capable neurons differentiated from stem cell cultures. External loading of JPW3027 is extremely simple and fast, and can be used to pre-select cells for further electrophysiological studies or to monitor the appearance and development of electrogenesis with minimal negative effect on culture health. This method has considerable advantages in comparison to commonly used Ca^2+^-indicators as a functionality assay for newly-differentiated neurons in culture. Firstly, the loading process is far simpler than the application of AM esters. Secondly, the dye can be used to monitor membrane potential not only in cell bodies, as is the case with most of AM dyes, but also in cellular processes. Thirdly, the VSDs used in this study report changes in membrane potential at the sub-millisecond range while Ca^2+^ indicators are several orders of magnitude slower. Fourthly, cultures can be re-used for further experiments after the loading process. And lastly, the voltage-sensitive dye can also be used to study spatial aspects of AP generation and propagation as well as electrical compartmentalization within the young neurons.

Multipotency and self-renewal abilities make stem cells the most promising source for cell replacement therapy in neurological disorders such as Parkinson's and Alzheimer's disease. However, one of the main issues in stem cell-based replacement therapy in neurology is to assess whether functional neurons are the final product of stem cell differentiation [10]. In several instances, cells can look like neurons but have electrical behaviors that indicate otherwise (Fig. 3) [10], [18]. Ultimately, the regenerative electrical activity defines a neuron and hence, only electrophysiology (including ion/potential imaging techniques) can prove that a differentiated cell is in fact a functional neuron. Here, we describe a simple and fast assay that can answer the differentiation question: Can the cell fire action potentials or not? Classic electrophysiological techniques are not trivial. They require specialized material and personnel that may not be available in molecular biology labs. Hence, dynamic imaging has been commonly used to assess neuronal functionality in differentiated cultures, primarily using Ca^2+^-sensitive dyes [3], [19] Ca^2+^ indicators can be delivered to neurons either by single cell injection (electroporation: [20]; whole cell patch-clamp: [21]) or bulk loading [22]. Except when the Ca^2+^ indicator is delivered locally using pressure ejection [23], the loading process of cultures takes longer than one hour [22] and can alter the physiological properties of the tissue [24]. The labeling of cells with JPW3027 on the other hand side takes between 5 and 10 minutes, does not require cell-toxic detergents (e.g. DMSO) and does not cause a substantial loss of cells on the coverslip compared to related approaches. In addition, we have not observed significant changes in membrane properties such as resting potential, input resistance and capacitance. The short loading process does not translate to poorer labeling. Cell bodies and processes are remarkably well marked by JPW3027 with very little spurious labeling (Fig. 1). Actually, bath application of JPW3027 (originally engineered for intracellular application) produced less background than RH795, a VSD engineered for extracellular application. VSDs bind indiscriminately to any lipid membrane [25] and we believe that the higher hydrophobicity of JPW3027 compared to RH795 causes a lesser affinity to lipids, producing a ‘cleaner’ labeling. While few studies so far have used VSDs to look at dissociated cells they commonly have been used in monitoring neuronal activity in slice preparations and in vivo [26].

While Ca^2+^ indicators can prove the presence of APs, the temporal resolution of these dyes does not permit to distinguish between mature and immature spikes based on AP-duration [6]. In fact, while there is a relationship between [Ca^2+^] and the number of APs in a burst it is not possible to resolve the timing of single APs within bursts [16]. Moreover, changes in [Ca^2+^] are not linearly related to membrane potential variation and diverse Ca^2+^ buffering properties of different sub-cellular compartments further complicate the relationship between [Ca^2+^] and membrane potential. On the other hand, several VSDs are capable to resolve changes in membrane potential with a linear variation in fluorescence [25] and in the sub-millisecond range [6], [27].

Obviously, the low exposure necessary in fast imaging decreases the amount of photons that hit the imaging sensor during each sweep, requiring more excitation light, which is associated with more cell damage. Also, VSD changes in Δ*F*/*F*_0_ in response to an AP are at least an order of magnitude smaller than changes reported by Ca^2+^ indicators [20], [25]. In order to improve the signal-to-noise ratio, other groups have averaged several image acquisition sweeps [27]. However, using an EM-CCD camera, we have shown that it is possible to separate spikes in the fluorescence signal with very low excitation light (minimizing cell damage) from the background noise based on single sweeps only. The back-illuminated EM-CCD camera used in this study has several important features for voltage imaging: high frame rate, large well size and a quantum efficiency that approaches 100%. While a high-end EM-CCD camera is a considerable aid to voltage imaging, cheaper camera options may be sufficient for AP detection in cultures using stimulation repetitions, signal processing and de-noising like ridge-tracking in frequency-time transforms [28].

The simplicity and time effectiveness of the VSD loading protocol makes the usage of potentiometric dyes ideal for functionality assays of culture dishes during different periods of development. For example, the same cell population could be assessed on different days in cultures plated on gridded coverslips [29] by reloading the culture with VSD each time an experiment is performed. Other methods like microelectrode array recordings could also be used to detect electrical activity in cell ensembles in stem cell cultures [30]. However, even using complex triangulation algorithms, it is not trivial to find the exact neuron that is firing in response to a stimulus in crowded cultures based on extracellular recordings from microelectrode arrays. Besides, even in sparse cultures, these arrays cannot provide information about the sub-cellular locus in which the electric activity is being generated. In fact, programmed compartmentalization of electrical activity is another major feature that distinguishes neurons from other excitable cells. The migration and anchoring of Na^+^ channels to the initial segment of the axon has been associated to several physiological processes [31]. In addition, AP-propagation is a fundamental function of a neuron and the manner in which an AP propagates is a strong indicator of maturation [32]. The high speed of voltage reporting of VSDs could also permit the study of AP-initiation and propagation in differentiated stem cell cultures as even subtle variations in AP timing like AP-initiation and propagation through different cell compartments can be picked up by VSD fluorometry [27]. For example, other groups have used intracellular JPW3027 dye to study AP initiation in layer 5 pyramidal neurons and demonstrate experimentally the saltatory propagation of APs through nodes of Ranvier [27].

To simplify the detection of firing-capable cells within stem cell cultures, stimulating electrodes could be positioned around a cell group to produce AP-triggering depolarization of the membrane. For time lapse experiments however, the stimulus has to be weak enough to not cause cell damage but strong enough to produce an AP in every cell in the area of interest. In a monolayer, the change in potential across the membrane produced by an extracellular stimulus can be well approximated by the finite element method (calculates the electric potential across the electrodes) [33], [34] and the cable equation (calculates the current across the membrane produced by the extracellular variation in electric potential) [14]. Our finite element model (Fig. 5B, C) was capable of reproducing the effects of a changing cathode position on membrane potential (Fig. 4B, C). Using the finite element method, it is possible to optimize the positioning of the stimulation anode and cathode to produce changes in extracellular potential that do not disturb cells away from the field of view (FOV). A further improvement on the neuron detection method would be software that automatically vectorizes micrographs, produces the meshes in the vectorized images and solves the partial Laplace equations for the FOV to determine the minimum voltage to be used for extracellular stimulation.

In conclusion, we demonstrate that voltage imaging is a versatile and simple method to detect differentiated neurons in stem cell cultures. Fast imaging technology is in constant advance; detectors are becoming faster and cheaper and new advances such as the scientific CMOS (or sCMOS) cameras can combine high-resolution and speed (using subfield scanning) [35]. Hence, using the same camera, relatively slow phenomena like Ca^2+^ signals or protein trafficking could be imaged in high spatial resolution while fast events like APs could be imaged with high temporal resolution. Also, the same methodology shown here could be applied to cultures pre-transfected with genetically-encoded voltage probes [36].

## Supporting Information

Movie S1 Fluorescence response of a cultured hippocampal neuron loaded externally with JPW3027. Time-lapse video showing changes in fluorescence triggered by an electrical stimulus. The video is played 20x slower than the original event and has been looped 6 times.
(3.11 MB MOV)
